# The role of haematological indices in predicting early iron deficiency among pregnant women in an urban area of Sri Lanka

**DOI:** 10.1186/s12878-018-0131-2

**Published:** 2018-12-22

**Authors:** Miruna Sudharshani Kalaimani Rabindrakumar, V. Pujitha Wickramasinghe, Lallindra Gooneratne, Carukshi Arambepola, Hemantha Senanayake, Tharanga Thoradeniya

**Affiliations:** 1 Department of Biochemistry and Molecular Biology, Faculty of Medicine, University of Colombo, Colombo, Sri Lanka; 2 Department of Paediatrics, Faculty of Medicine, University of Colombo, Colombo, Sri Lanka; 3 Department of Pathology, Faculty of Medicine, University of Colombo, Colombo, Sri Lanka; 4 Department of Community Medicine, Faculty of Medicine, University of Colombo, Colombo, Sri Lanka; 5 Department of Obstetrics and Gynecology, Faculty of Medicine, University of Colombo, Colombo, Sri Lanka

**Keywords:** Iron deficiency, Haemoglobin, Red cell indices, Pregnancy

## Abstract

**Background:**

Early detection and treatment of iron deficiency during pregnancy is crucial for optimum pregnancy outcomes. Anaemia is a late indictor of iron deficiency measured as Hb < 11 g/dL, and is widely used as a proxy for iron deficiency. We aimed to evaluate the role of red cell indices as a screening tool for early detection of iron deficiency among pregnant women in an urban area of Sri Lanka.

**Method:**

A cross-sectional study was conducted among 110 apparently healthy pregnant women ≤12 weeks of gestation attending antenatal clinics in Colombo, Sri Lanka. Women already on nutritional supplements were excluded. Full blood count, serum ferritin (SF) and high sensitive C-reactive protein (hs-CRP) assessments were performed. The women with evidence of inflammation as indicated by hs-CRP > 10 mg/L were excluded (*N* = 20) from data analysis. Anaemia (Hb < 11 g/dL) and iron deficiency (SF < 30 μg/L) were defined according to WHO guidelines. Receiver operating characteristics curves were used to derive red blood cell indices that showed the optimal cut-offs in detecting early iron deficiency.

**Results:**

Of the 90 women, 63 (70.0%) were iron deficient (SF < 30 μg/L), out of whom 10 (15.9%) were identified as having iron deficiency anaemia (Hb < 11 g/dL). A high sensitivity (> 70%) in the prediction of iron deficiency was obtained for the optimal cut-off values of Hb < 12.2 g/dL, MCV < 83.2 fl, MCH < 26.9 pg and MCHC 33.2 g/dL while maintaining a specificity > 40%.

**Conclusion:**

Iron deficiency can be predicted in early stages using Hb and red cell indices, which is much less expensive. This could be a useful method in areas with limited resources and a high prevalence of iron deficiency.

## Background

Iron deficiency is the commonest nutritional problem in the world, affecting over two billion people. Iron deficiency anaemia is its main consequence, considered a global public health problem, mostly affecting developing countries. Pregnant women, women of child bearing age and young children are the most vulnerable to develop iron deficiency anaemia. It is a well-recognized direct and indirect cause of maternal death [1]. Globally, 38% of pregnant women are affected by anaemia, mostly due to iron deficiency [1–3].

Iron deficiency results when iron stores become depleted due to long-standing negative iron balance. This reduces ‘storage iron’ such as ferritin and haemosiderin, in turn affecting the normal iron turnover in the body and compromises the supply of iron to the transport protein apotransferrin [4]. This leads to decreased transferrin saturation and increased transferrin receptor availability in the circulation, leaving no more iron to be mobilized if additional iron is required [4, 5]. If not corrected, it will shift to the second stage known as iron deficient erythropoiesis where haemoglobin (Hb) concentration starts to fall due to impaired red blood cell synthesis, resulting in iron deficiency anaemia. Correspondingly, it causes a detectable change in the mean corpuscular haemoglobin (MCH), mean corpuscular haemoglobin concentration (MCHC) and mean corpuscular volume (MCV) [4, 5].

Iron deficiency is recognized to lead to adverse pregnancy outcomes such as intra-uterine growth retardation, low birth weight, preterm birth, and perinatal death [6]. Association of iron deficiency with impaired foetal growth, poor cognitive performance and behaviour in infants is well established [7–9]. Recent evidence suggests that iron deficiency during peri-conceptional period and the first trimester could have a profound and long term effect on the brain development of the infant, even at levels of iron deficiency that is not sufficient to cause maternal anaemia [10]. Further, maternal iron deficiency is a risk factor for iron deficiency in infants [11, 12]. Studies in animal models have shown that peri-conceptional and early life iron deficiency alters dopamine metabolism, myelination and development of the hippocampus with concurrent impairment of brain development [13, 14]. Although some changes are reversible, some early insults of iron deficiency would be permanent [13, 14]. Therefore, the British guidelines on the management of iron deficiency in pregnancy recommend targeting prevention of early iron deficiency indicated as serum ferritin (SF) < 30 μg/L during first trimester [15–17].

To prevent and control iron deficiency and iron deficiency anaemia and its adverse maternal outcomes, WHO has developed iron deficiency prevention programmes and recommends universal iron replacement [18]. The routine practice in Sri Lankan antenatal clinics is to measure the Hb concentration of pregnant women at the booking visit and at 28 weeks of gestation [19, 20]. Women identified with anaemia (Hb < 11 g/dL) at booking visit and at 28 weeks are treated with 120 mg of elemental iron daily while others are placed on a supplemental dose of 60 mg of elemental iron throughout pregnancy [18–20].

The gold standard for assessing iron deficiency is bone marrow analysis, which is too invasive as a screening tool in the routine clinical practice [21]. Serum ferritin, serum transferrin and serum iron are good markers to diagnose iron deficiency [5, 15] but these too are relatively expensive assays. Compared to these, red cell indices that are a component of the full blood count determined by automated haematology analysers are less invasive, less cumbersome and inexpensive.

Therefore, we aimed to evaluate the validity of each red cell index including Hb as a screening tool to detect early iron deficiency at an early stage of pregnancy.

## Methods

### Study design

A cross-sectional study was conducted among 110 apparently healthy pregnant women of ≤12 weeks of gestation attending antenatal clinics of the Medical Officer of Health (MOH) of the Colombo Municipal Council areas from September 2015 to March 2016. Only women whose period of gestation (POG) was verified as being ≤12 weeks by ultrasound scanning were recruited. Women already on nutritional supplements; diagnosed with long standing illness or on medication that are known to interfere with micronutrient metabolism (e.g. antiepileptic drugs such as acetazolamide, carbamazepine and clobazam, Aspirin and Antacids containing Magnesium hydroxide); with a past history of pregnancy-associated complications (e.g. pre-eclampsia or gestational diabetes mellitus), multiple pregnancy; suspected of having congenital malformations in the fetus and with evidence of infection (identified by c-reactive protein (CRP) > 10 mg/L [22]; those diagnosed with or having a family history of thalassemia and other haematological disorders were excluded. The sample size was calculated [23] to test the diagnostic accuracy of red cell indices to identify iron deficiency in pregnancy. The assumed areas under the receiver operating characteristic (ROC) curve of red cell indices in the diagnosis of iron deficiency were based on a previous study (Hb: 0.95, MCV: 0.79, MCH: 0.82, MCHC: 0.88, mean red blood cell distribution width (RDW): 0.78 and red blood cells (RBC): 0.69) [24]. Using a given marginal error of 0.1 and 95% confidence interval [23], the sample size was 84. To compensate for attrition and exclusions due to concurrent infection, 25% was added and the final sample size was 110. The study was approved by the Ethics Review Committee, Faculty of Medicine, University of Colombo, Sri Lanka. Approval was obtained from the Colombo Municipal Council and the MOH at the antenatal clinics and informed written consent was taken from study participants.

### Data collection

An interviewer-administered questionnaire was used to obtain the sociodemographic information of participants. Antenatal records were used to verify the POG, parity, contraceptive method used, and past obstetric history of the participants. Maternal anthropometry was measured using a standard protocol. Weight was recorded to the nearest 0.1 kg using a weighing scale (Salter, England; 145 BKDR) and height was measured to the nearest 1 cm using a stadiometer (Seca, UK; 225). Non-fasting peripheral venous blood samples were collected under sterile conditions into EDTA (1 ml) tubes and to tubes without anticoagulant for serum separation. Blood samples were transported to the laboratory in ice, and serum was separated within 3 h of collection.

### Laboratory analysis

Measurements of Hb, MCV, MCH, MCHC, RDW, RBC and packed cell volume (PCV) were obtained by haematology analyser; Celltac E-Nihon Kohden Corp, Tokyo from an accredited laboratory. Serum ferritin was measured by Enzyme Linked Immunosorbent Assays (ELISA) using reagent kits (Cat No.160116 & Diagnostic Automation, Inc., USA). C-reactive protein was measured by sensitive immunoturbidometric assay by Dimension® high sensitivity C-reactive protein (hs-CRP) method (Cat No-434 & Dimension RxL Max, Siemens, USA). Experimental standards were maintained by daily calibration with standard external quality control (QC) material (Cat No. 370 & Lyphochek immunoassay plus control, Bio RAD, USA) and pooled serum analysis as an internal quality control. The inter-assay coefficient of variance (CV) and intra-assay CV were used to check the precision and maintained within 3SD of QC values. According to the WHO guidelines, anemia was defined as Hb < 11 g/dl [25]; and iron deficiency as SF < 30 μg/l [16, 17]. Out of 110 women, 20 with hs- CRP > 10 mg/l were considered as harboring an inflammatory state [22] and excluded from the analysis. The remaining 90 women were categorized as having deficient or sufficient iron stores based on the recommended cut-off for serum ferritin (30 μg/dL).

### Statistical analysis

Statistical analysis was performed using statistical software SPSS version 21 (SPSS Inc., Chicago, IL). One-sample Kolmogorov-Smirnov analysis was used to check the normality of the variables. Concentrations of Hb, MCV, MCH, MCHC, RBC and PCV were normally distributed. Non-normally distributed data (SF and RDW) were log transformed and antilogarithms were used to present the relevant data. Descriptive and chi square tests were used to describe the sociodemographic characteristics of the women. Correlations between SF and red cell indices (Hb, MCV, MCH, MCHC, RDW and RBC) were examined by Pearson’s correlation coefficients. The mean concentration of red cell indices were compared using Student *t* test between iron deficient (SF < 30 μg/dL) and women with sufficient iron stores (SF ≥ 30 μg/dL). Statistical significance was set at *p* value less than 0.05.

Receiver operating characteristic curves were constructed to identify the optimal cut-off for red cell indices (Hb, MCV, MCH and MCHC) with the most effective measure of sensitivity and specificity in diagnosing iron deficiency during early pregnancy. The red cell indices were compared against the true iron deficiency indicated by SF < 30 μg/dL. Youden’s Index was calculated as (sensitivity + specificity) – 100 using the ROC curves.

## Results

The mean age was 26.0 years while the mean POG at booking visit was 9 weeks. Approximately 31% of the women were primigravida. Most of the women (78.8%) were from low socio-economic status, with a monthly family income of less than USD 294. Nearly, 88% of women had secondary education and 84% were unemployed. The general characteristics of these women according to their iron status are shown in Table 1. There were no significant differences in the socio demographic characteristics between iron deficient and iron sufficient women.

**Table 1:** General characteristics according to their iron status at ≤ 12 weeks of gestation

Characteristics	All subjects (*N* = 90)	Women with iron deficiency SF < 30 μg/L (*N* = 63)^a^	Women with sufficient iron stores SF ≥30 μg/L (*N* = 27) ^a^	*p-value*
Age in years (Mean ± SD	26 ± 5	26 ± 5	26 ± 5	0.28
POG in weeks (Mean + SD)	9 ± 2	9 ± 2	9 ± 2	0.38
Gravidity, *n* (%)				
Prime	28 (31.1)	22 (34.9)	06 (22.2)	0.41
One	34(37.8)	23 (36.5)	11 (40.7)	
≥ two	28 (31.1)	18 (28.6)	10 (37.1)	
Ethnicity, n (%)				
Sinhala	28 (31.1)	18 (28.6)	10 (37.0)	0.70
Tamil	27 (30.0)	19 (30.2)	08 (29.6)	
Moor	33 (36.7)	24 (38.1)	09 (33.4)	
Other	02 (2.2)	02 (3.1)	00 (0.0)	
Level of education, n (%)				
No schooling	04 (4.4)	02 (3.2)	02 (7.4)	0.22
Primary	04 (4.4)	04 (6.3)	00	
Secondary	79 (87.8)	56 (88.9)	23 (85.2)	
Collegiate	03 (3.4)	01 (1.6)	02 (7.4)	
Tertiary	00	00	00	
Employment, n (%)				
Unemployed	76 (84.4)	54 (85.7)	22 (81.5)	0.21
Employed	14 (15.6)	09 (14.3)	05 (18.5)	
Monthly family income, n (%)				
< USD 294	71 (78.8)	51 (81.0)	20 (74.1)	0.32
≥ USD 294	14 (15.6)	10 (15.9)	04 (14.8)	
Don’t know	05 (5.6)	02 (3.1)	03 (11.1)	

*Abbreviations: SF* serum ferritin, *N* number of pregnant women, *SD* standard deviation, *POG* period of gestation, *n* (*%*) number (percentage)

^a^Data on SF concentration available for 90 women after excluding CRP > 10 mg/L

Of the 90 women, 13 (14.4%) were anaemic (Hb < 11 g/dL) while 63 (70%) were iron deficient (SF < 30 μg/L) during early pregnancy. Among the 63 iron deficient women 10 had iron deficiency anaemia (Hb < 11 g/dL and SF < 30 μg/L). The remaining 53 women were not anemic (Hb > 11 g/dL). The concentrations of Hb, MCV, MCH and MCHC were significantly lower (*P* < 0.05) in the iron deficient than the iron sufficient women (Table 2). A significant positive correlation was observed between SF and Hb (*r* = 0.358; *p* = 0.001), MCV (*r* = 0.322; *p* = 0.002), MCH (*r* = 0.333; *p* = 0.001) and MCHC (*r* = 0.286; *p* = 0.006). No significant association was observed between SF and RDW and RBC.

**Table 2:** Distribution of red cell indices according to the iron status of pregnant women (*N* = 90)

Red cell indices	Women with iron deficiency SF < 30 μg/L *N* = 63 *Mean ± SD*	Women with sufficient iron stores SF ≥30 μg/L *N* = 27 *Mean ± SD*	*p* value
Hb (g/dL)	11.9 ± 1.1	12.4 ± 1.0	0.038^*^
MCV (fl)	82.2 ± 6.6	85.2 ± 4.8	0.028^*^
MCH (pg)	26.7 ± 1.1	28.1 ± 1.1	0.016^*^
MCHC (g/dl)	32.7 ± 0.9	33.1 ± 0.7	0.040^*^
RDW (%)^a^	12.6 ± 1.1	12.3 ± 1.0	0.197
RBC (× 10^9^/L)	4.4 ± 0.3	4.3 ± 0.5	0.245
PCV (%)	36.4 ± 2.9	37.2 ± 3.0	0.218

*Abbreviations: N* number of pregnant women, *SD* standard deviation, *Hb* haemoglobin, *MCV* mean corpuscular volume, *MCH* mean corpuscular haemoglobin, *MCHC* mean corpuscular haemoglobin concentration, *RDW* red blood cell distribution width and *RBC* red blood cells

^*^ *Significantly different (p < 0.05)*

^a^ *Geometric mean*

**Fig. 1:**
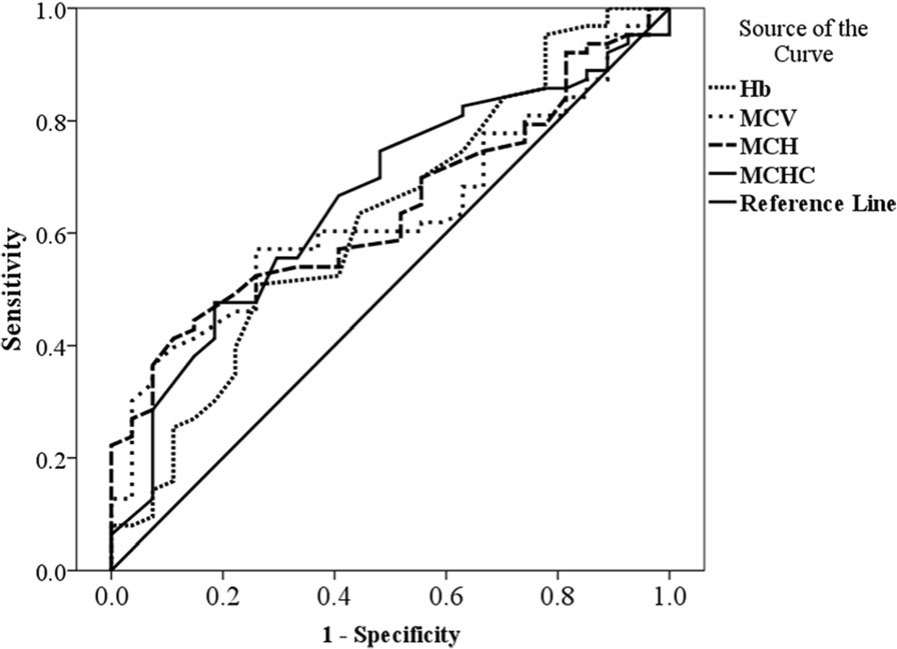
Receiver operating characteristic curves of red cell indices in the prediction of early iron deficiency in early pregnancy. The ROC curves of Hb, MCV, MCH and MCHC were compared in the prediction of early iron deficiency indicated by Serum ferritin < 30 μg/dL. *Abbreviations: Hb* haemoglobin, *MCV* mean corpuscular volume, *MCH* mean corpuscular haemoglobin, *MCHC* mean corpuscular haemoglobin concentration

Receiver operating characteristics curves were constructed for red cell indices to identify optimal cut-offs to predict iron deficiency (Fig. 1). Table 3 shows the diagnostic performance of red cell indices with optimal cut-off acquired via ROC curves. Haemoglobin concentration of < 12.2 g/dL was found to be an optimal cut-off to detect early iron deficiency providing a sensitivity of 74.1 and 50.8% specificity. Similarly, > 70% sensitivity was noted for the optimal cut-offs of MCV < 83.2 fl, MCH < 26.9 pg and MCHC 33.2 g/dL in the prediction of early iron deficiency while maintaining the specificity > 40%.

**Table 3:** Diagnostic performance of red cell indices in the prediction of iron deficiency at early pregnancy

Red cell Indices	Area under the ROC curve	*p* value	Optimal cut-off	Sensitivity(%)	Specificity (%)
Hb (g/dL)	0.625	0.062	12.2	74.1	50.8
MCV (fl)	0.631	0.05	83.2	74.1	57.1
MCH (pg)	0.642	0.034	26.9	88.9	41.3
MCHC (g/dl)	0.659	0.017	33.2	74.6	51.9

*Abbreviations:%* percentage, *Hb* haemoglobin, *MCV* mean corpuscular volume, *MCH* mean corpuscular haemoglobin, *MCHC* mean corpuscular haemoglobin concentration

## Discussion

The present study noted that although only 14% (13/90) of studied women at early pregnancy are anaemic (Hb < 11 g/dL), a significant number of 63/90 (70%) are iron deficient as assessed by SF. The percentage of anaemia noted in our study is similar to the values reported in the national nutrition survey in Sri Lanka in 2009 and in other smaller studies conducted in Sri Lanka [26–29].

Serum ferritin measurements provide a reliable indication of early iron deficiency during pregnancy [15, 16]. A SF concentration < 15 μg/dL indicates depleted iron stores [17] and nearly 46% of women in the present study had depleted iron stores. Studies conducted in Sri Lanka have shown that 40–50% of women have depleted iron stores during their reproductive age and are at risk of developing anemia during pregnancy [30, 31].

Importantly, the present study showed that iron deficient women are not captured through routine antenatal care by way of Hb measurement. The women identified to be anaemic through routine antenatal care (Hb < 11 g/dL) were provided with double dose of iron supplementation (120 mg elemental iron per day) and were followed up to monitor the improvement in Hb levels. Special advice on iron-rich foods and adhering to supplements is also given to these women. The non-anaemic – iron deficient women go undetected and do not receive special care, thus are at high risk of becoming anaemic later in pregnancy. In addition, their infants are at higher risk of developing cognitive impairment and behavioral abnormalities [32].

Low Hb concentration is not a specific indicator of iron deficiency and studies have highlighted the poor diagnostic performance of Hb in the prediction of iron deficiency [33, 34]. Considering the magnitude of the problem the current study is a preliminary effort to determine the role of red cell indices in screening for iron deficiency in Sri Lankan pregnant women. Van den Broek et al., [16] have shown that the cut-off point of 30 μg/L of SF concentration is a better indicator of low iron stores in pregnant women following validation with gold standard assessment of bone marrow iron content. World Health Organization (WHO) guidelines recommend the same cut off (< 30 μg/L) to detect iron deficiency in populations with chronic inflammation such as in population with malaria and other infections. [17]. Moreover, the recent British guidelines [15] indicates that the treatment should start when the SF concentration is < 30 μg/L, even if the woman is non-anaemic as detected by Hb. Therefore, the current study used SF 30 μg/L as a gold standard to define early stage of iron deficiency. Although the British guideline mentions serum ferritin as a screening tool, it recommends using it on targeted populations rather than universally, considering the time and its cost effectiveness [15]. In a resource-poor setting like Sri Lanka a simple cost effective surrogate for serum ferritin would be of greater value.

The ROC curves were constructed to identify optimal cut-off values for red cell indices to detect iron deficiency in early pregnancy. Most of the countries supplement pregnant women with iron based on the Hb concentration and assume that they are iron deficient. It is necessary and crucial to correctly identify the truly iron deficient women who have normal Hb values. Therefore, our goal was to achieve a high sensitivity while maintaining an effective specificity.

The Hb concentration of 12.2 g/dL was selected as an optimal cut-off for the detection of iron deficiency in early pregnancy. This value is considerably higher than the WHO recommended cut-off (Hb = 11 g/dL) for anaemia in women during early pregnancy [25]. Similarly, a study conducted among Mexican pregnant women demonstrated a Hb concentration of 11.5 g/dL as predictive of iron deficiency during late pregnancy [34]. When considering the other red cell indices, there are no recognized cut-off values for MCV, MCH and MCHC during pregnancy. The present study derived optimal cut-offs for these red cell indices with a high sensitivity (> 70%) while maintaining a greater than 40% specificity. A study conducted among Indian women suggested optimal cut-offs for Hb; 9.7 g/dL, MCV; 76.1 fl, MCH; 25.05 pg and MCHC; 31.35 g/dL in predicting iron deficiency anaemia during second and third trimesters [24]. The optimum cut-off derived for MCV in the present study is higher than the cut-off (80 fl) widely used to identify microcytic anaemia which is caused by iron deficiency [35]. This may be due to the increase in MCV during pregnancy [36]. Further, a higher area under the ROC curves were noted for MCHC (0.659) and MCH (0.642) when compared to Hb (0.625) and MCV (0.631). Based on the ROC curves the sensitivity of MCHC and the MCH were 74.6 and 88.9% reaching for women with iron deficiency respectively.

## Conclusion

The present study confirms the unsuitability of the recommended cut-off for Hb as a screening tool for iron deficiency in early pregnancy. The high rates of false negative results preventing iron deficient women from receiving appropriate treatment. The results could be precarious in places with high iron deficiency rates as observed in the present study. Iron deficiency could be predicted using hematological indices that are produced when a full blood count is ordered. This is a much less expensive test that could of great value in areas with limited resources and a high burden. Although this study has given promising results, iron replacement and vigilance on the improvement of serum ferritin and these red cell indices will enhance the clinical interpretations. Further, it may be appropriate to conduct a study on a larger population across different socio economic strata.

## Abbreviations

CV: Co-efficient of variance
Hb: Haemoglobin
hs-CRP: High sensitivity C-reactive protein
LRMP: Last regular menstrual period
MCH: Mean corpuscular haemoglobin
MCHC: Mean corpuscular haemoglobin concentration
MCV: Mean corpuscular volume
PCV: Packed cell volume
POA: Period of amenorrhoea
POG: Period of gestation
QC: Quality control
RBC: Red blood cells
RDW: Red blood cell distribution width
ROC: Receiver operating characteristic
SD: Standard deviation
WHO: World Health Organization
